# PB.23: Effect of detector type on cancer detection in digital mammography

**DOI:** 10.1186/bcr3523

**Published:** 2013-11-08

**Authors:** A Mackenzie, LM Warren, DR Dance, J Cooke, M Halling-Brown, P Looney, DP Chakraborty, MG Wallis, RM Given-Wilson, KC Young

**Affiliations:** 1 National Co-ordinating Centre for the Physics of Mammography, Royal Surrey County Hospital NHS Foundation Trust, Guildford, UK; 2 Department of Physics, University of Surrey, Guildford, UK; 3 Jarvis Breast Screening and Diagnostic Centre, Guildford, UK; 4 Scientific Computing, Medical Physics, Royal Surrey County Hospital NHS Foundation Trust, Guildford, UK; 5 Department of Radiology, University of Pittsburgh, PA, USA; 6 Cambridge Breast Unit, Cambridge University Hospitals NHS Foundation Trust, Cambridge, UK; 7 NIHR Cambridge Biomedical Research Centre, Cambridge, UK; 8 Department of Radiology, St George's Healthcare NHS Trust, London, UK

## Introduction

This work measured the effect that image quality associated with different detectors has on cancer detection in mammography using a novel method for changing the appearance of images.

## Methods

A set of 270 mammography cases (one view, both breasts) was acquired using five Hologic Selenias and two Hologic Dimensions X-ray units: 80 normal, 80 with simulated inserted subtle calcification clusters, 80 with subtle real noncalcification malignant lesions and 30 with benign lesions (biopsy proven). These 270 cases (Arm 1) were converted to appear as if they had been acquired on two other imaging systems: needle image plate computed radiography (CR) (Arm 2) and powder phosphor CR (Arm 3). Three experienced mammography readers marked the location of suspected cancers in the images and classified whether each lesion would require further investigation and the confidence in that decision. Performance was calculated as the area under curve (AUC) of the alternative free-response receiver operating characteristic curve.

## Results

The AUCs of Arms 1 to 3 were 0.750, 0.707 and 0.648 for calcification clusters and 0.744, 0.722 and 0.694 for noncalcification cancers. The differences between all of the arms for calcification clusters were statistically significant (*P *< 0.05). No significant differences were found for noncalcification lesions. The percentage of correctly marked lesions that were classified as recalled lesions dropped from 58% to 39% (calcifications) and from 72% to 62% (non-calcifications) between Arms 1 and 3.

## Conclusion

Detector type has a significant impact on the detection and recall of calcification clusters but not noncalcification cancers.

